# Unraveling the medical residency selection game

**DOI:** 10.1007/s10459-020-09982-x

**Published:** 2020-09-01

**Authors:** Lokke M. Gennissen, Karen M. Stegers-Jager, Jacqueline de Graaf, Cornelia R. M. G. Fluit, Matthijs de Hoog

**Affiliations:** 1 Institute of Medical Education Research Rotterdam (iMERR), Room Ae-227, Erasmus MC, Postbus 2040, 3000 CA Rotterdam, The Netherlands; 2 Radboud UMC, Nijmegen, The Netherlands; 3 Department of Internal Medicine at Radboud UMC, Nijmegen, The Netherlands; 4 Center on Research in Learning and Education, Radboud University Medical Center Health Academy, Nijmegen, The Netherlands; 5 Department of Pediatrics at Erasmus MC, Rotterdam, The Netherlands

**Keywords:** Diversity, Residency selection, Specialty training, Post-graduate medical education, Qualitative research

## Abstract

The diversity of modern society is often not represented in the medical workforce. This might be partly due to selection practices. We need to better understand decision-making processes by selection committees in order to improve selection procedures with regard to diversity. This paper reports on a qualitative study with a socio-constructivist perspective conducted in 2015 that explored how residency selection decision-making occurred within four specialties in two regions in the Netherlands. Data included transcripts of the decision-making meetings and of one-on-one interviews with committee members before and after the group decision-making meetings. Candidates struggled to portray themselves favorably as they had to balance playing by the rules and being authentic; between fitting in and standing out. Although admissions committees had a welcoming stance to diversity, their practices were unintentionally preventing them from hiring underrepresented minority (URM) candidates. While negotiating admissions is difficult for all candidates, it is presumably even more complicated for URM candidates. This seems to be having a negative influence on attaining workforce diversity. Current beliefs, which make committees mistakenly feel they are acting fairly, might actually justify biased practices. Awareness of the role of committee members in these processes is an essential first step.

**Electronic supplementary material:**

The online version of this article (10.1007/s10459-020-09982-x) contains supplementary material, which is available to authorized users.

## Introduction

The increasing sociocultural diversity in Western society is underrepresented in the medical workforce (Leyerzapf and Abma 2012; Verdonk and Janczukowicz 2018), partly because we seem to lose medical graduates with a sociocultural diverse background, i.e. underrepresented minorities (URM) in the transition to residency (Leyerzapf and Abma 2012). This is unfortunate in light of the increasing demand for culturally sensitive care (Saha et al. 2008; Goldsmith 2000; U.S. Department of Health Human Services 2006; Verdonk and Janczukowicz 2018; Cohen et al. 2002).

Diversity stimulation is thought to incorporate the knowledge and skills of minority talents in healthcare and at the same time to increase the cultural sensitivity of the majority of physicians (Cohen et al. 2002; Nivet 2015). Furthermore, it is essential from a social justice point of view to have equal career opportunities for all within the medical field (Thomas and Ely 1996).

Despite the increasing number of URM medical students in many Western countries, they continue to be underrepresented in residency training and in the medical specialist workforce (Leyerzapf et al. 2015; Verdonk and Janczukowicz 2018). In the Dutch context, there has been a slight increase in URM candidates among medical school attendants. The possible adverse impact of selection practices is receiving increasing attention both from researchers and from national organizations, including the Ministry of Health. Nevertheless, this increased attention has not been reflected in a formal policy regarding diversity. Not only are they generally underrepresented, the proportion of URM medical graduates appears to decrease along the career path to becoming a medical specialist (Leyerzapf and Abma 2012; Sopoaga et al. 2017). This phenomenon has been referred to as the ‘leaky pipeline’ (Freeman et al. 2016).

Much of selection research (Patterson et al. 2016; Roberts et al. 2017) has conceptualized admissions decision-making as a step-by-step cognitive process that produces an optimal decision (Bolander and Sandberg 2013). Therefore, research has focused on how to optimally measure candidates’ characteristics or knowledge and to determine which attributes are predictive for future performance. Two recent reviews suggest that some selection tools are more likely to have adverse impact on URM candidates than others (Patterson et al. 2016; Stegers-Jager 2018). However, the reported studies generally do not consider the social context in which selection takes place, thereby assuming selection committees are a static non-contributing factor.

As gatekeepers, selection committees determine what they recognize and prioritize as relevant information and eventually how they combine and interpret this information for the purpose of decision making. This is relevant in medical school selection, and probably even more so in postgraduate selection where the number of candidates is smaller, committees are smaller, and usually all candidates are interviewed by all committee members.

Previous research in selection for medical school, has examined mechanisms in selection practices which potentially hinder diversity. Razack and colleagues analyzed Canadian policy documents concerning medical school student selection, identifying a strong meritocratic belief that “the best and the brightest” should enter the medical profession (Razack et al. 2015). This belief seems to overrule all other arguments, including equity and service-to-society. Wright (2015) evaluated the preparation of students in the UK for medical school selection by focusing on the support of high schools in the selection procedure and found considerable differences in support between private and state schools. Although these studies reflect contexts with hundreds of candidates and at an earlier stage in the medical career pipeline, these mechanisms could also be relevant in the postgraduate medical setting (Wright 2015).

In this postgraduate medical setting, research that takes the context of the selection committee into account is lacking. Leyerzapf et al. (2015) and Woolf et al. (2016) probably go closest while explaining differences in attainment of postgraduate training positions. Leyerzapf and colleagues analyzed performance appraisals in daily medical practice and revealed an apparent disadvantage for URM medical doctors based on implicit criteria, such as whether one fits into the team and whether one has the right social network (Leyerzapf et al. 2015). Woolf and colleagues explored the perceptions of minority and majority health care professionals on causes of differential attainment in UK postgraduate medical training. They identified additional difficulties faced by UK minority and international graduates impeding their learning and performance in postgraduate training (Woolf et al. 2016).

The decision-making practices of medical residency selection committees have, to the best of our knowledge, not yet been subject of research. Besides, there is little empirical evidence looking at selection in practice. Therefore, the purpose of this paper is to gain insight in this practice with particular focus on potential unintended excluding mechanisms for URM candidates.

## Methods

### Approach

The design of this qualitative study was explorative and inductive, considering the scarcity of knowledge regarding residency selection decision-making processes. We used a socio-constructivist perspective, approaching the decision-making process as a social construct, shaped by interpretations and meaning giving of the professionals involved.

### Context/setting

This study was conducted in the Netherlands, where medical school training is 6 years. A majority of medical graduates gains work experience as a resident-not-in-training before applying for residency training. This is particularly relevant for getting into highly competitive residency programs. The design of the selection procedure for residency training is controlled by the national association of that particular specialty and by the regional residency program groups, resulting in a great diversity in actual practices of selection procedures.

### Sampling

We purposively sampled six divergent selection procedures, of which all selection committee members were invited to participate. Sampling continued until adding new procedures did not lead to new insights or additional information regarding the research purpose.

### Procedure/design

To get rich and concrete data actual selection procedures were used. As is common in the Netherlands, the selection committees in the six studied procedures applied a traditional job interview setting. First, a preselection based on candidates’ motivation letters and résumés was made. Next, job interviews were held with the selected candidates. Finally, selection committee members decide in a group discussion which candidates to hire. One of the selection procedures additionally contained an assessment round. The research design is described according to this general setting. We introduced rankings in the procedure as we assumed that consciously ranking the candidates during the procedure could help committee members to recall decisions or unveil unconscious steps in the decision making process. The selection committee members were asked to rank the candidates numerically according to their individual preference at three moments: (1) after they received the motivation letter and résumés; (2) after the job interviews; and (3) after the group discussion. Two separate one-on-one semi structured interviews were held with all selection committee members. The first interview took place after the preselection of the candidates, but before the job interviews and the second interview took place after the whole procedure. Additionally, the decision-making group discussions were observed and recorded.

We combined interviews, rankings and observations in the analysis since triangulation of these data sources enabled a more detailed and balanced view on the process.

### Interviews

The one-on-one interviews enabled us to explore the individual views of the committee members on the selection process and provided safety for the participants to express their own view without hierarchical influences. All interviews were conducted by LG. A general interview guide was used for the core of the semi-structured interviews (“Appendix”), but adjustments were allowed for new insights, emerging topics of interest or divergent cases.

### Selection decision-making group discussion

The video and audio recordings of the group discussions were set up to least interfere with the group decision-making process. The same researcher who conducted the interviews (LG), observed the group discussions. In total, this study includes 7.5 h of observation. Observational notes were made and worked up into detailed descriptions shortly after. Interviews and group discussion recordings were transcribed verbatim.

### Analysis

Data were analyzed by three researchers LG (female white Dutch Ph.D. student with a medical background), KSJ (female white Dutch post-doctoral educational researcher with expertise on diversity and selection in medical school) and LF (female white Dutch MD, post-doctoral educational researcher with expertise on workplace learning in health education).

Transcripts were analyzed following the template analysis approach developed by King, entailing a thematic analysis using a constructed coding template (King and Horrocks 2010). Given its importance in respect to the research aims ‘diversity’ was an a priori theme. Following the initial coding process, a template was developed. This template was further developed in subsequent coding and continuously critically reviewed on accordance with the data.

The data were coded by LG and if necessary, discussed and reviewed by KSJ and/or LF. The findings were regularly reviewed and discussed with the two other researchers (JdG, MdH), both involved in resident selection procedures. Their perspective enriched our understanding of the process, by affirming our findings and challenging us to justify our decisions. Throughout the study we regularly considered to what extent our backgrounds, perspectives and possible biases influenced the research process. To keep the construction of the template transparent an audit trail was produced. Results were fed back to research participants in order to further optimize our understanding. AtlasTi 7.5 was used to manage the analysis.

### Analytic framework

We assume that the decision-making process is a dynamic and emergent social process, shaped by a negotiation of interpretations and meaning giving of the professionals involved. Selection committee members seek to incorporate the information gathered about candidates to make informed decisions. Therefore, the way of receiving, processing and prioritizing information is a key element in the selection process. We argue that by the way they handle the information, selection committees construct what they recognize as ‘merit’. Similar to Posselt’s (2016) reasoning, we approached merit as a relative measure rather than an absolute measure of achievements to date and perceived potential. Merit is assessed in comparison with the other candidates in the selection procedure, or even with candidates from previous and future selection procedures. As such merit is an admissibility assessment relative to a specific candidate pool. It also depends on the composition of the selection committee, composed of members with specific personal preferences.

Following this reasoning, Posselt infers that there is not a clear single hierarchy of admission criteria. Therefore, it is important to understand selection within the context of the selection committees (Posselt 2016).

The main focus in our study was the actual decision process with specific attention towards mechanisms that unintentionally hinder diversity. Instead of putting explicit focus on diversity in our interviews, we explored what selection committees recognize and prioritize as relevant information to assess merit and how they eventually combine and interpret this information for the purpose of decision making. Thus, we aimed at identifying mechanisms in these processes which could exclude URM candidates.

As noted previously, we had one a priori code which was labelled diversity. We started with open coding, which resulted in a more extensive template than necessary to answer our research question. Next, we continued with those elements of the template needed for answering our specific research question. The three overarching themes arising from the data were (1) image creation, (2) decision-making of the selection committees, and (3) the complex route towards gaining entrance to residency for candidates. Zooming in further on these themes revealed relevant areas of tension. Additionally, we kept diversity as a separate theme.

## Results

The selection procedures in the present study all concerned hospital based specialties. Committees’ compositions are displayed in Table 1. Five of the six committees were fully composed of physicians within that specialty, i.e. either medical specialists or residents. One selection committee included a human resource management specialist. A total of 75 interviews and seven group discussions were analyzed. For the sake of confidentiality, the descriptive information disclosed is limited.

**Table 1:** Selection committee composition of included selection procedures

	Specialty	Size of committee	F/M ratio^a^	Residents	Candidates
Selection committee 1	A1	8	3/5	2	6
Selection committee 2	A1	8	3/5	2	17
Selection committee 3	B	8	1/7	1	13
Selection committee 4	C	8	2/6	1	10
Selection committee 5	D	5	2/3	1	6
Selection committee 6	A2	8	4/4	2	10
		9	4/5	2	6

^a^Ratio of female/male selection committee members

Our data resulted in a complex template representing a diversity of themes involved in the process of residency selection decision-making. Below, we present themes which in our perspective reveal how the selection committees negotiated the candidates’ merit. This is relevant to understand since selection committees need to get a clear view on the candidates’ experience, skills, motivation and personality characteristics in order to be able to identify the best possible candidate. In this process selection committee members need to negotiate a hierarchy in these criteria. The hierarchy reveals how merit is constructed by this particular selection committee. We considered three themes as most relevant for our research goal and most illustrative of possible constraints for diversity. The themes are interrelated, but for the sake of clarity, we describe them separately.

Illustrative quotations from the interviews or observations are presented in Table 2.

**Table 2:** Illustrative quotations per theme

Playing by the rules of the game: being visible	“For they’ve already put themselves on the radar, they were a resident-not-in-training here or organized an introductory interview or, so I’ve seen all of them once or several times before”	Interview before job interviews Committee 1 Selection committee member 1
Playing by the rules of the game: being visible	“The residents, erm especially the *hospital A* residents they know her and I happen to know her because of my internship here, but in *hospital B* and in hospital C* and nobody knows her. At the regional get-togethers and activities, she is sort of mousy, she’s been called that, but the residents who know her in *hospital A* do support her”	Group discussion Committee 3 Selection committee member 9
Playing by the rules of the game: an interest of selection committee members	“Well, you know that’s what makes it an interesting workplace here for young doctors. If people can move on from here into the training program, then they would like to come and work here. And that means I am offered better people and that I get a better work climate. A work climate that is a little more competitive, more focused on quality. If I don’t get any residents or just selected from somewhere else and here they would be….. Then my residents-not-in-training are and it would do them no good”	Interview after job interviews Committee 4 Selection committee member 8
Being authentic	“Behavior, how somebody sits and if he’s comfortable presenting himself in an honest way, is open”	Interview before job interviews Committee 1 Selection committee member 3
	“You just miss something in the replies and the authenticity somebody radiates, then something is just not right”	Interview before job interviews Committee 1 Selection committee member 3
	“Yeah, yeah. And then he may say he really wants it, but still it doesn’t feel, it still doesn’t feel that way. Something is just not right”	Interview before job interviews Committee 4 Selection committee member 1
	“No but also in the preparation. She didn’t know the research program either. Erm, you see it’s these kind of things all the time, like: “Just, just not right.” So perhaps…”	Group discussion Committee 5 Selection committee member 2
	“They’re all rehearsed answers, just not what I’m looking for. You can’t beat it out of them even if you really put the pressure on…. Now tell me how things really are, then they’re afraid to tell, it’s just a show and a show doesn’t do much good”	Interview after job interviews Committee 3 Selection committee member 5
Standing out	“And coming back to that, anyway another plus factor could be something entirely different; so someone who has really done something novel. That…who really…. that’s what I think. Just done something almost nobody has done before. So take that girl with her own company, that’s a plus factor in my book. Somebody who studied something entirely different from Maths and Science. You know, things like that…I can. That is why this APPL2, who takes on something completely different, yes I think so too”	Interview after job interviews Committee 1 Selection committee member 2
	“Putting one’s head above the parapet”	Interview after job interviews Committee 1 Selection committee member 6
	“Stand out”	Interview before job interviews Committee 1 Selection committee member 1
Fitting in	“And now we should take a look, who fits in here, who can start on the first of January and who do we think is good enough to be admitted.”	Interview after job interviews Committee 5 Selection committee member 2
	“-Program director: Does that fit in with the group?	Group discussion Committee 2 Program director and resident
	-Resident: Yes. I think so	
	-Program director: You’re very careful	
	-Resident: Yes. No. She really is different. But I… I think you can take the chance. Yes. That … that … that chance. Yes”	
	“We also value the opinion of the residents. So we’ll hear the residents’ opinion in the group”	Interview before job interviews Committee 3 Selection committee member 7
	“Comes across as reliable. Someone you can have a pint with, friendly, fairly stable impression, shall I put it that way”	Interview after job interviews Committee 3 Selection committee member 4
Diversity	“With applications I very much look at, erm, diversity, that is I don’t want uniformity, we don’t need uniformity at all in the Netherlands… … … of course we have # residents here and the atmosphere in such a group is extremely important and erm, whoever you take on will have an impact, and I won’t say it’s the decisive factor but at the end of the day it might decide the choice between 2 candidates, if there’s not much between them otherwise you might opt for a candidate who you think will add to the team and that diversity, we talked about this before that is the diversity I am looking for also in erm, ethnicity preferably, if at all possible, and that is well, that is complicated”	Interview before job interviews Committee 1 Selection committee member 1
Condition of equal suitability	“So the funny thing is the selection committee we’re all men. Well that’s not really a good thing, is it, most of the time we do have one or two women here that was and not now and the people we take on are mostly women … … and I always say, we choose the best one but in the case of equal suitability and so on. That is why the other thing I think is important, especially here is that you keep your eyes open for cultural diversity. Hey, we’ve all got white blonde women and we’ve got 1 or 2 with a color and last time we took on a Moroccan girl. Without a headscarf to be sure, but she … and then with equal suitability. That again is something that also plays a part in your selection”	Interview after job interviews Selection committee 1 Selection committee member 6
Attribution to candidate	“No, not a lot, No not a lot in fact and that’s funny really …. Especially here at the basic training some 50%, rough estimate, is of a different origin and we don’t really see them show up here. For whatever reason. I haven’t quite figured it out, so that’s not a lot. …. Sometimes with the residents you do see of course for they’re the same ones. And still you pretty rarely see a clear ambition to move on then to our specialism and taking action to facilitate that. …Or they still prefer GP medicine. I don’t know, if other subjects … But quite a few are clumsy or are lagging behind in some way or don’t fight hard enough but in any case quite often they’re already rejected because of their CVs and things like that. At any rate they’re not as good as others”-	Interview after job interviews Selection committee 1 Selection committee member 6

### Theme 1: Being authentic yet playing by the rules of the game

Most of the selection committee members highly valued authenticity; that is, candidates showing honesty and vulnerability in the job interview. Yet, being too honest might have a negative impact since candidates ought to play by the rules of the game. Besides explicit rules, the ‘selection game’ also knows implicit and tacit rules that candidates are expected to be familiar with and adhere to. One implicit rule is to expose yourself in the right way. This crucial self-representation actually already starts before the official selection procedure and might be achieved in different ways in the different specialties. Commonly, visibility is achieved through clerkships and or previous (clinical) work or research, which enable candidates to be seen and known by the selection committee members. A more specific strategy is putting oneself on the radar by planning an informative meeting with the program director before applying or by attending activities organized by and for the regional residents.

Another implicit rule refers to the specific road taken towards a certain specialty; some roads are less questioned than others. This road, which often starts before finishing medical school, refers to CV building activities of a candidate. As an example, candidates should, be aware of the benefits of work experience in a teaching hospital: it is in the interest of selection committee members to get the candidate who has worked, or still works, at their institution into residency training.

Furthermore, although most selection committee members claim to value an honest answer over the ‘right’ answer, some answers seem to be more desirable. Candidates have to navigate through a labyrinth by being authentic at the right times, yet being mindful to keep playing by the rules of the game. A similar mechanism is seen in the expected level of preparation. Most selection committee members expect the candidates to be prepared for questions of the job interview and disapprove of candidates who come across as not well prepared. Yet selection committee members dislike rehearsed answers, because it makes them question candidates’ authenticity. Some selection committee members help the candidates they are acquainted with (e.g. candidates working in their hospitals) in preparing the job interview.

### Theme 2: Standing out yet fitting in

Selecting the most desirable candidate out of a collection of talented candidates is a demanding task. Selection committee members recognize that the abundance of suitable candidates has raised their expectations of candidates over the years, a mechanism similar to credential-inflation. Selection committees look for competencies or characteristics that set candidates apart from the others, which most often is considered a good thing. Nevertheless, they highly value candidates that fit in the group. So ideally, a candidate fits in, yet stands out of the group as well. Standing out is conceptualized as possessing distinctive characteristics as well as being an added value to the existent residency group or, on a bigger scale, to the specialty. Standing out may manifest itself in an interesting profile or originality in the application letter, resume or job interview without being far-fetched or coming across as unauthentic.

Fitting in is conceptualized as fitting the culture of a particular residency program at several levels. First of all, selection committees are looking for candidates with whom they have a “click”. Particular activities of candidates resonate more with selection committees than others. Secondly, fitting in is conceptualized as fitting in the existing group of residents. This aspect is strongly influenced by residents in the selection committee. Remarkably, fitting in seemed just as important in the committee with a sociocultural diverse residency group as in the other committees.

Standing out is not easy, given the abundance of well qualified candidates. Selection committee members seem to be aware of the rising bar for entering their specialty. Still, their highest priority remains selecting the most excellent candidate. Both person and doctor features are assessed in the selection procedures. Interestingly, in their negotiation of merit selection committees tend to focus more on what candidates have to offer next to being a good doctor. Having the potential to become a good medical specialist seems to be a prerequisite, but not sufficient to obtain a residency position. One of the arguments raised to reject a candidate was: “If somebody has four years of clinical experience, he should at least have something extra besides that. I don’t know what extra he’s got besides that, no CV…” (Group discussion)

### Theme 3: Stance towards diversity

When asked what was important for them in selecting candidates, committee members frequently mentioned diversity, both in candidates’ backgrounds and ambitions.

They see the additional value of diversity in the dynamics and balance of the group, and seem aware of the societal need for a diversity of doctors.

“With applications I very much look at, erm, diversity; that is, I don’t want uniformity, we don’t need uniformity at all in the Netherlands…. … ….. of course we have # residents here and the atmosphere in such a group is extremely important and, erm, whoever you take on will have an impact, and I won’t say it’s the decisive factor but at the end of the day it might decide the choice between two candidates, if there’s not much between them otherwise you might opt for a candidate who you think will add to the team. And this diversity, we talked about this before, is the diversity I am looking for also in, erm, ethnicity preferably, if at all possible, and that is well, that is complicated.” As soon as diversity is discussed, the atmosphere in the group is mentioned as counterbalance, which is claimed to be extremely important. The strong tendency to look for someone who will fit in the group is likely to hinder diversity, since fit is often based on similarity. Selection committees appear cautious in taking risks regarding fitting in. By contrasting diversity and fitting in the group, they seem to perceive diversity as a potential risk for fitting in.

Remarkably, almost all committee members who mentioned the importance of diversity added a condition of equal suitability which has to be met in order to be willing to select the more diverse candidate. A committee member elaborates: “So the funny thing is, the selection committee we’re all men. Well that’s not really a good thing, is it, most of the time we do have one or two women here that was and not now and the people we take on are mostly women … … and I always say, we choose the best one but in the case of equal suitability and so on. That is why the other thing I think is important, especially here is that you keep your eyes open for cultural diversity. Hey, we’ve all got white blond women and we’ve got one or two with a color and last time we took on a Moroccan girl. Without a headscarf to be sure, but she … and then with equal suitability. That again is something that also plays a part in your selection.”

In this quote it becomes obvious that diversity is only seen as an added value if all other aspects are considered equal in merit. Choosing the best seems to be their first priority and only when there is no one “best” candidate, diversity will be considered. The “best” appears to be defined as the one having the most merit, regardless of a candidate’s historic context or privileges, which might further advantage those already privileged.

Nevertheless, this quote shows that there are URM medical doctors who make it into the medical specialties. The question remains whether and to what extent these URM medical doctors had to adjust to get in, thereby possibly decreasing their potentially added values.

Some selection committees noticed that only few URM candidates applied for their selection procedures, which was commonly attributed to different career preferences of these candidates. Although other explanations were offered for the lower number of URM candidates, the general tendency was to hold candidates accountable. A selection committee member said: “No, not a lot, no not a lot in fact and that’s funny really …. Especially here at the basic training some 50%, rough estimate, is of a different origin and we don’t really see them show up here. For whatever reason. I haven’t quite figured it out, so that’s not a lot. …. Sometimes with the residents you do see of course for they’re the same ones. And still you pretty rarely see a clear ambition to move on then to our specialty and taking action to facilitate that. …Or they still prefer GP medicine. I don’t know, if other subjects ….. But quite a few are clumsy or are lagging behind in some way or don’t fight hard enough. But in any case, quite often they’re already rejected because of their CVs and things like that. At any rate they’re not as good as others.” This selection committee member emphasizes the need to have a clear ambition and to already take action to realize it during medical school. While some of these actions may be quite explicit, those which will help along the road to a certain specialty are often implicit. In order to get to know these implicit rules, candidates need social capital; someone who will explain the rules of the game or show them the way.

## Discussion

In this explorative study, we studied the decision-making process of residency selection committees in actual practice, including potential, unintended, additional barriers for URM candidates. We showed that candidates struggle to portray themselves favorably as they have to balance between playing the game and being authentic, and between fitting in and standing out. Candidates are expected to behave authentically in interviews, meanwhile playing by the implicit rules of the game. Additionally, candidates are expected to stand out of the candidate pool regarding their added value to the current residency group, yet they should also fit in this group. The abundance of candidates creates a mechanism of credential inflation with regard to the selection criteria. Selection committee members are looking for excellence, and often construct excellence in person features rather than doctor features.

We also highlighted unintended mechanisms that may lead to additional hurdles for URM candidates, while balancing between playing the game and being authentic, and between fitting in and standing out. As mentioned before, fitting in has the pitfall to be highly based on similarity (Rivera 2012). Furthermore, playing the game by the rules may be more problematic for URM candidates, since the rules are implicit and often require social capital to become familiar with them. Finally, differences in privilege may exist; candidates may have an advantage over others on account of being member of a particular social category. Selection committees claim to look for diversity, but at the same time their practices and procedures seem to hinder them in actually hiring URM candidates.

In our study, committee members apparently fall prey to similarity bias (McPherson et al. 2001; Orpen 1984). In their attempt to select the best candidate, selection committees are actually selecting candidates whose choices they can understand, whom they find interesting, or who seem like pleasant colleagues and fit the group best. This could be seen as risk-aversive behaviour in the high-stakes decision of selecting for residency. They choose candidates whose behavior they can presumably predict because these candidates think, feel and act very similarly to themselves. Given the many candidates with the potential to become a good doctor, hiring decisions are rarely based on task-related criteria. This leaves more room for other arguments, which might perpetuate similarity bias.

The findings of our study suggest that the meritocratic discourse identified by Razack et al. (2015) is also present in postgraduate medical education. Currently, the most important goal of selection seems to be selecting “the best”, which might contribute to or maintain the current workforce imbalance. Selecting the best seems to foster favoring high ambitions besides patient care (e.g. having ambitions in research or management), whereas we would argue that in healthcare the aim of selection should be to create a medical workforce that fulfils societal needs. An excessive focus on high ambitions in research or management might lead to overselecting future doctors for jobs in academia and large hospitals, where these ambitions can easier be fulfilled. This potentially adds to the current shortage of medical specialists in smaller hospitals, especially in rural areas (Rabinowitz et al. 2008).

Another remarkable finding is that selection committee members’ beliefs of being fair and non-discriminatory might actually justify inequitable practices. They try to weigh the candidates based on their negotiated construction of merit, and choose the best on the basis of equal criteria. By implicitly taking merit as a static, absolute, and fair assessment, they bounce the responsibility for unequal outcomes back to the candidates. In fact, the admission procedure might disadvantage certain groups if potential barriers on their career paths are not taken into account. The most desirable career path towards residency, built on contextualised ideas of merit and excellence, is implicit and tacit and thus only known to “insiders” (Razack et al. 2015). Social connections, and thus one’s social capital, are crucial to become “an insider” or to gain inside information (Bourdieu 1986). Although this phenomenon has not yet been described in the postgraduate medical setting, it is not unique. Similar results have been found in different contexts, such as academia and in medical school selection (Van den Brink 2010; Razack et al. 2015; Wright 2015).

An important practical implication of our study is that selection committees should be aware that measuring all candidates to the same yardstick might actually not be that fair. Selecting from an equity point of view requires a more holistic and contextualized approach, in which we acknowledge differences in chances in the career path so far. Simultaneously, attention should be given to inclusiveness and belonging along the whole career path of a medical doctor. We will never enjoy diversity to its full potential, when URM doctors feel that they do not belong or should compromise their authenticity in order to belong (Sherbin and Rashid 2017).

This study shows how selection committees unconsciously construct barriers in their selection procedures, which keep them from including URM talents in our medical workforce. This phenomenon calls for a critical revision of our notion of selection as a step-by-step process of uncovering candidates’ characteristics and knowledge leading to an ‘optimal and unbiased decision’. The process and the selectors themselves are important actors in this phenomenon. We therefore argue that besides the valuable psychometric research on this matter, we should look more in-depth at how selection committee members (unconsciously) raise barriers through their construction of merit. Based on such analyses interventions could be designed, focused on shifting the goal of selection from selecting the arbitrary best towards selecting for the workforce.

Our qualitative design allowed us to look at a considerable amount of empirical data, with different sources of information allowing triangulation. With respect to the transferability of our findings, the identified practices ultimately must be understood in the context of postgraduate selection in the Netherlands. With the aim to provide the reader with the necessary information to be able to evaluate the transferability and relevance of this work to other contexts, we provided a detailed description of the Dutch context and discussed our findings in the light of previous literature. Some limitations should be taken into account. First, we only included hospital-based competitive specialties. Although we consciously chose for these specialties, this might question transferability to selection procedures of less competitive specialties. Second, although our strength is that we included actual practices and our priority was to have a minimal influence on these practices, it cannot be ruled out that, for example, the presence of a researcher at the group discussion influenced the practice.

## Conclusion

Based on our study of residency selection decision-making in the Netherlands, we argue that selection committees, led by their dedication to look for the best candidates, construct merit as nearly impossible combinations of good qualities. Paradoxical combinations and the fuzzy and tacit career map make it difficult for all candidates to obtain a residency position. Probably, it is even more complicated for URM candidates to convey themselves as the desired candidate. Interventions at the level of selection alone are unlikely to bring about the desired change, particularly if these are solely focused on postgraduate selection and ignore the other leakages along the pipeline. Simultaneous interventions towards inclusivity in medical school and postgraduate medical education are crucial in enabling the desired change. For selection committees, interventions could be sought in developing more awareness of the crucial roles of committee members in the selection decision making processes, having critical dialogues on what is perceived as merit and critically evaluating the purpose of selection. Research on selection should be extended with research on how current selection tools are actually used in decision-making. Our advice is to look for unintentional barriers for diversity in our current selection decision making practices.

### Electronic supplementary material

Below is the link to the electronic supplementary material.

Supplementary material 1 (DOC 142 kb)

## Appendix: Interview guide

### Before the job interviews

- Interviewer introduces herself and short explanation study
- Could you introduce yourself and tell me something about your daily tasks and your role in the selection?
- Do you have an image of the desired endpoint (future medical specialist) in mind which you want to select for in this specific selection round?
- Can you describe what kind of profile you are looking for?
- How would you describe your own profile?
- Which selection criteria do you use while selecting?
- Which information do you need from the formal selection procedure to be able to judge a applicant for desirability for hiring?
- Which information do you use or need from informal sources?
- Do you miss information with regard to applicants?
- Discuss the pre-ranking with the selection committee member:
  - Can you explain this ranking for me? E.g. Why is the first applicant the most desirable of this group applicants at this stage of the selection procedure? Why is the applicant at the bottom least desirable from this applicant group?

### After job interviews

- Which information did you use in judging the desirability of this candidate?
- Which information from the informal circuit did you use or find useful in the selection decision-making?
- Which information is still lacking, which in your opinion might enhance decision-making when it’s available in selection decision making? Why is this information important in your opinion?
- To what extent does your preference or ranking correspond with the eventual decisions made in the selection procedure? (If not, what happened in the group discussion? E.g. To what extend did you feel your opinion was heard?)
- Discuss the rankings (pre-/during/after) with the selection committee member:
  - Can you explain this ranking for me?
  - Can you explain the shifts in the rankings? (e.g. Can you explain what happened which made this candidate shift from a top position down to the middle section?)
