# Supplementary material for: Unraveling the medical residency selection game
Source: Adv Health Sci Educ Theory Pract. 2020 Sep 1;26(1):237–52. doi: 10.1007/s10459-020-09982-x (PMC7900052; doi:10.1007/s10459-020-09982-x)

**Summary of Analytic Steps with regard to template:**

We started our template with one a-priori set code: diversity. We treated this a-priori set code just as any of the template codes as tentative, and if necessary, subject to redefinition or removal when needed to optimally fit to the data. The first version of the template was inductively established, since it was based on inductive line by line coding of a sub-set of transcripts (14 interviews + 1 group discussion). The coding, as well as categorizing and organizing of these codes was done by three researchers (LG,CF, KSJ). After establishing a first template, we discussed the template with MdH and JdG, to triangulate with their experience from practice and we asked them to challenge us actively to explain how and why we came to this template based on the data.

*TEMPLATE 1:*

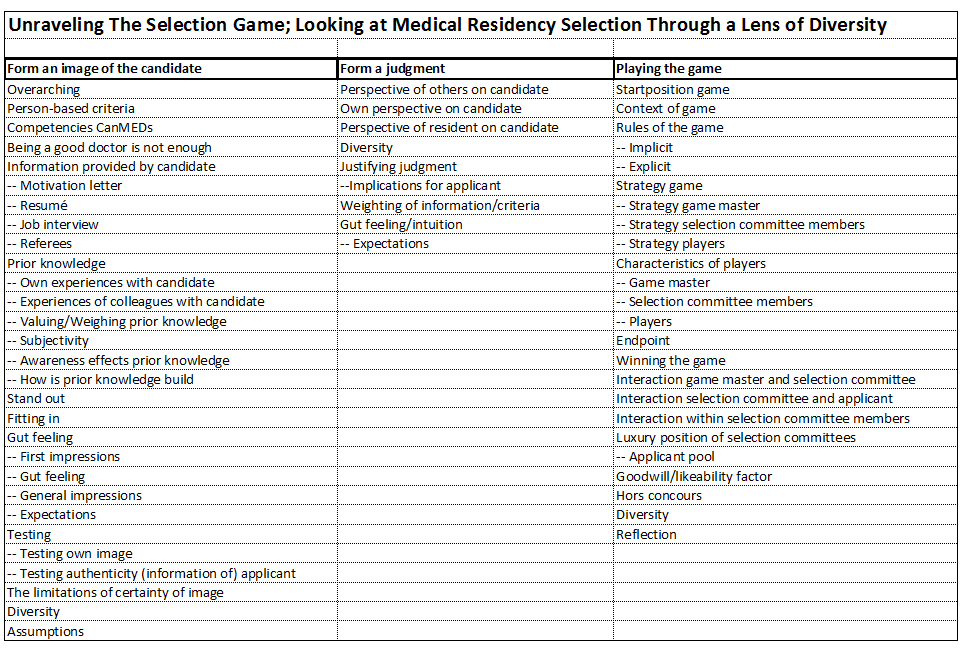


Since we noticed that this would be a more extensive template then strictly necessary to answer our research question, the three researchers (LG,KSJ, CF) coded another sub-set of transcript line by line (13 interviews + 1 group discussion), and discussed the two following questions afterwards. First and foremost, whether our extensive template held for this data as well. We argued which codes needed to be redefined or removed to hold for this selection round as well. Second, we discussed which elements are relevant to answer our research question and should be maintained in further versions of the template. Based on the existing template two perspectives were found most relevant in the data with regard to our research goal (i.e. to gain insight in the decision-making process of a residency selection committee in actual practice and to explore if there are additional hurdles for sociocultural diverse candidates). First, a perspective in which relevant themes of the image creation and judgment-making of the selection committees can be placed. Second, a perspective in which relevant themes with regard to the complex route towards gaining entrance to residency for an applicant could be placed. After defining these themes as first level codes, we discussed which second-level themes were relevant, and noticed some interesting areas of tension. We focused our template to the areas of tension that would help us answering our research question. This led to three areas of tension under ‘form an image and judgement’. For the second perspective covering the complex route towards gaining residency entrance we categorized themes which were relevant as preparation long before the selection procedure started (i.e. what kind of things a candidate should start doing in medical school in order to start sorting for a specialty) separately -which we refer to as ‘the leap necessary for the obstacle course’ in the template- from themes related to the actual selection procedure (i.e. preparation for the job interview) –which we refer to as ‘selection procedure’ in the template. Here we included areas of tension which we found in the data between certain themes.

*TEMPLATE 2:*
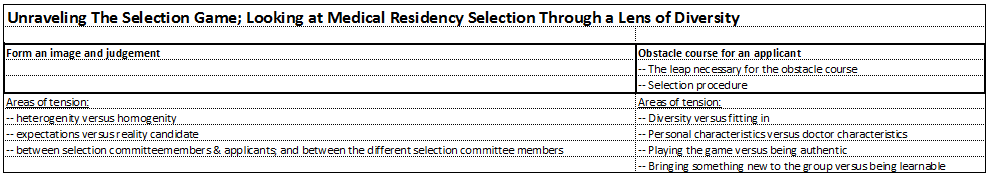

LG continued coding with this template, in which areas of tension were coded by separately coding the two ends of the areas of tension for instance: ‘diversity’ and ‘fitting in’. LG coded the remaining data based on this template and any questionable bits and pieces were discussed frequently with CF and KSJ. Based on these discussions we developed a clear hierarchical template with general themes and areas of tensions.

*TEMPLATE 3:*
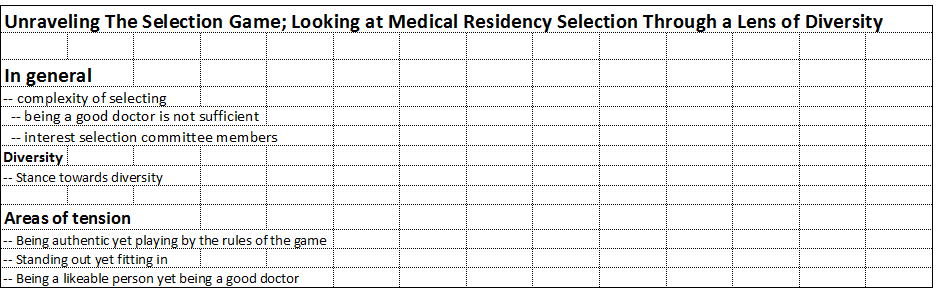


After coding all the data, the next step was to triangulate the findings by presenting these results back to the different selection committee members (participants), to research peers and at conferences. All groups recognized the themes, and gave us input to further reorganize the template to be able to present our results in a more concise and clear way. We maintained two areas of tension and integrated the third one (being a likeable person versus being a good doctor) and the ‘in general’ themes in these two areas of tension. We decided to frame the areas of tension by … yet …, rather than the earlier chosen … versus …, because of a closer fit. Diversity also remained a separate first level theme, although one could see an area of tension in this theme as well (lip service yet practices which raise additional hurdles).

*TEMPLATE 4:*

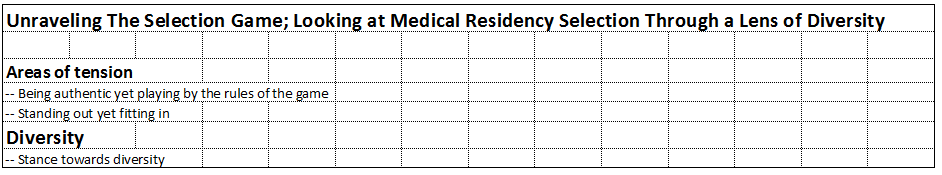

Supplement: Supplementary file 1 — Supplementary material 1 (DOC 142 kb) [file 10459_2020_9982_MOESM1_ESM.doc]
